# Domains for global health education and practice

**DOI:** 10.1007/s40037-013-0067-y

**Published:** 2013-07-09

**Authors:** Daryl Ramai

**Affiliations:** School of Medicine, St. George’s University, True Blue, Grenada, West Indies

Dear Sir,

As world economies become ever more globalized and interconnected, physicians are faced with new opportunities and challenges. Travel and immigration between continents raises concerns of communicable diseases and multidrug-resistant microorganisms. Occurrences in a separate region can no longer be ignored, but must be taken into profound consideration. To this end, ‘Tomorrow’s Doctors’ must be prepared with a newly revised set of skills, and a philosophy where students realize that there is more than the Western paradigm of diseases and health care.

In the United States, the number of medical students acquiring global health experience continues to grow [1]. Some medical schools have recognized the necessity and value of having global health electives or courses in their curricular. For these future doctors, the benefits of acquiring international health experience can have far-reaching consequences. Haq et al. [2] report that medical students are able to more readily recognize the signs and symptoms of a disease, improve their comprehensive physical examination skills, and acquire greater cultural sensitivity.

However, the work load of a rigorous medical school curriculum may inhibit some students from taking an additional course or optional elective overseas. Universities may not be poised to intercalate an additional requirement on their students. Therefore, how are we to address the concept of global health and develop competency amongst medical students? To this end, Houpt et al. [3] have outlined three domains to be incorporated into a modern curricular. Firstly, medical students should have a broader understanding of the major diseases affecting developing countries. This knowledge serves to inform students on what type of care is needed to address travellers and immigrants. It also provides students with an understanding of the barriers affecting health care systems in developing parts of the world.

Secondly, while the body of knowledge related to travel medicine is vast and volatile, a modern curricular should concentrate on a core set of relevant topics. The American Committee on Clinical Tropical Medicine and Traveller’s Health emphasizes that faculty dedicate (a median of) 30 h to teaching topics on malaria, tropical disease clinical syndromes, global health epidemiology, global HIV, tuberculosis, and intestinal protozoa. The final recommendation involves a growing immigrant population which brings the risk of foreign diseases and its impact on native health. Hoput et al. use the example of tuberculosis where it is reported that 53.3 % of cases are foreign-born patients. Consequently, medical students must be guided on how to provide holistic and comprehensive care to this expanding immigrant population to ensure proper cultural sensitivity.

In conclusion, while an additional course may not be practical for some medical students or institutions, the incorporation of the aforementioned domains may serve as a platform towards achieving competency in global health delivery. This revised paradigm would ultimately shift attention to a more comprehensive understanding of medicine and health care in the world.
